# Supplementary material for: Shiga Toxin-Producing E. coli (STEC) from Danish Patients, 1997–2023: Diagnostic Trends and Bacteriological Findings
Source: Microorganisms. 2025 Oct 12;13(10):2342. doi: 10.3390/microorganisms13102342 (PMC12565805; doi:10.3390/microorganisms13102342)
Supplement: Supplementary file 1 [file microorganisms-13-02342-s001.zip › Supplementary material DK STEC 1997-2023.pdf]

Supplementary material

Characterisation the national reference laboratory at SSI

Methods and procedures

Initial processing of faecal specimens: Approximately 0.1 g faecal material was stirred in 2 mL of sterile buffered saline (80 mM NaCl, 50 mM Na_2HPO_4, 10mM KH_2PO_4, pH 7.38). An aliquot (ca. 10 µl) of the saline suspension was plated on SSI enteric medium (SSI; Cat. No. 785) (1) and examined for the presence of *Salmonella, Yersinia, Vibrios* and *Shigella.* The faecal suspensions were furthermore examined for other enteropathogens including selected virus and parasites as requested specifically by the submitting physician.
Primary screening for diarrheagenic E. coli *using dot blots 1997-2007:* Bacterial cultures were plated directly on SSI enteric medium and examined for lactose- and non-lactose fermenting coliform colonies. One to three lactose- and/or non-lactose fermenting, morphologically different coliform colonies from the SSI enteric medium were seeded onto three separate nylon membranes (Hybond-N+, Kat. Nr.: RPN1732B, Amersham Pharmacia Biotech, UK) placed on non-selective agar plates. In some cases, and a sweep from an area with confluent growth was also included. Control of growth was performed on a fourth non-selective blood agar plate. Colony and sweep dot blots were hybridised with the following DNA probes; One pool of DNA probes for detection of STEC and enteroinvasive *E. coli* (EIEC) contained DNA probes derived from NTP705, Shiga toxin 1 (*stx1*) (2), DEP28, Shiga toxin 2 (*stx*2) (3) and WR390, the invasion plasmid antigen gene *ipaH* found in EIEC and *Shigella* (4) and a single probe for the detection of the *eae* gene, which encodes the adherence factor intimin, found in Enteropathogenic *E. coli* (EPEC), attaching and effacing *E. coli* (A/EEC) and STEC (5). A second pool for the detection of enterotoxigenic *E. coli* (ETEC) contained DNA probes for LT (*eltI*), ST_h (*estA*h) and ST_p (*estA*p) (6). The probes were labelled with Digoxigenin-d-UTP (Boehringer) by random priming and/or PCR as described previously (7). Bacterial culture blots were hybridised under stringent conditions according to the manufacturer's prescriptions.
Primary screening using DEC PCR 2007-2023: DNA from one to three lactose- and/or non-lactose fermenting, morphologically different coliform colonies from the SSI enteric medium were marked on the primary plate and then pooled and extracted in Chelex 100 in 10 mM Tris-HCl, 1 mM EDTA, pH 8. The DNA pools were subjected to gene detection by PCR for *stx*- and *eae* genes as described by Persson *et al.* (8).
Supplementary Analysis in Cases of Culture Failure: In cases where no bacterial culture could be isolated, a minimum of 50 µl extracted DNA from the primary diagnostic laboratory were submitted to SSI from specimens that were suspected to be part of an outbreak and from specimens from patients with HUS. The DNA was subjected to *stx* subtyping (9) and/or selected O group determination for eight O groups: O26, O103, O111, O121, O145, O146, O157 and O91 as described by Iguchi *et al.* (10).

Phenotypic and genotypic characterisation of the isolates:

Colony Recovery and Initial Confirmation:

Single colonies that reacted positively with the DNA probes were recovered from the primary SSI enteric plate (2007-2023) or the control blood agar plate (1997-2007). These isolates were confirmed for target gene presence and further characterised. In specimens where only the sweep

dot blot was positive, bacterial culture from a zone of dense growth on the primary plate was re-examined with the purpose of identifying colonies reacting with the probes or positive by DEC PCR.

Confirmation and Phenotypic Typing of E. coli Isolates
Prior to 2014, the strains were confirmed as being *E. coli* using the Minibact E kit (SSI; Cat. No. 905) (11) and tested for β-glucuronidase production on PGUA plates (SSI; Cat. No. 722) (12). Phenotypic O:H serotyping was done by microtiter plate- and tube agglutination using methods described previously (13, 14). See also Standard Operation Procedures for O & H serotyping and reference strains for a detailed description of phenotypic serotyping of *E. coli*. Production of Shiga toxin was examined using the Vero cell assay (VCA) as previously described (7). Haemolysin production was examined on blood agar plates using 5% defibrinated washed sheep blood (15).

Detection of Additional Virulence Genes:
Presence of additional virulence genes was examined using DNA probes derived from pSS126, the Enteroaggregative heat stable toxin (*astA*), CVD419, the plasmid encoded enterohaemolysin (*ehxA*) (16), PS2.5, the invasive plasmid in EIEC (17), CVD432, the plasmid marker for Enteroaggregative *E. coli* (EAEC) (18), and SLM862, detecting the *daaC* gene from the *daa*-locus encoding the afimbrial adhesin F1845, mediating diffuse adherence (DA) of *E. coli* (19). Presence of the STEC autoagglutinating adhesion gene (*saa*) was examined by PCR as described by Paton *et al.* (20). *stx* subtyping was done as described by Scheutz *et al.* (9) or using the *E. coli* plugin in BioNumerics (version 8.1.8, BioMérieux).

WGS-based methods:
From 2014, all STEC strains were whole genome sequenced (WGS) and analysed *in silico.*
WGS was performed at SSI, where DNA was extracted using DNeasy Blood and Tissue Kit (Qiagen, Copenhagen, Denmark) or MagNA Pure 96 DNA Multi-Sample Kit (Life Technologies, Carlsbad, CA, USA). Library preparation for short-read sequencing was performed using an Illumina Nextseq (Illumina, San Diego, CA, USA), to obtain paired-end reads of 2x250bp or 2x150bp. Quality control was performed of the sequences. Initially, QC was based on evaluation of genome size, number of contigs, N50 and average coverage 30x, species confirmation through KmerFinder (https://cge.food.dtu.dk/services/KmerFinder/). Since QC has been performed through the standardized in-house pipeline, Bifrost (github.com/ssi-dk/bifrost). Bifrost checks for proper genome size (4.6 to 5.6 megabase pairs), sufficient sequence data, proper number of contigs (< 500), and does contamination checks (allowing up to 5% of other genera). Furthermore, it does *de novo* assembly (SPADES) and assigns MLST. The *in silico* serotype and virulence genes were determined evaluating output from both an in-house standardized script using KMA mapping and from the output of the BioNumerics *E. coli* plugin - both based on gene detection from the VirulenceFinder and SerotypeFinder databases (https://cge.cbs.dtu.dk/services/) (21-24). In case of ambiguous or non-typeable *in silico* O group phenotypical O-typing was carried out.
Inconclusive subtyping was re-examined using additional mapping in CLC Genomics Workbench v10.
Strains that were phenotypically non-motile (H-) were assigned H-types *in silico,* and similarly the *in silico* O-group was used on phenotypically O rough and O?

Update of the CGE VirulenceFinder database and virulence profiles

The database at CGE was expanded to include an improved detection of 68 *eae* alleles found in *E. coli* and *E. albertii*, which were subdivided into seven subtypes: 21 *eae*-alpha, 8 *eae*-beta, 15 *eae*-epsilon, 10 *eae*- gamma, 4 *eae*-iota, 6 *eae*-lambda, and 4 *eae*-rho, see **T2** in supplementary material **(SM)** for accession numbers and translation of previously used designations of *eae* alleles. Addition of genes found in *Shigella* and EIEC included *icsA* (actin polymerization, 12 alleles), *ipaD* (invasion protein *Shigella flexneri*, 2 alleles), invasion plasmid antigen genes *ipaH*, (plasmid encoded, 3 alleles), *ipaH*7.8 (plasmid encoded, 11 alleles), *ipaH*9.8 (chromosomal, 14 alleles) and *lacY* (lactose permease, 74 alleles). Marker genes for the four modules on the 86-kb chromosomal mosaic element Locus of Adhesion and Autoaggregation (LAA) described by Montero *et al.* (25) included *sisA*, ability to attenuate the host inflammatory response induced by UPEC, *hes*, hemagglutinin from STEC (module I), *iha*, iron regulation, *nmpC*, heat resistance, *lesP*, LAA-encoded Serine Protease Autotransporter of *Enterobacteriaceae* (SPATE) (module II), *pagC*, serum resistance phenotype, *tpsA*, a two-partner secretion system that in Gram-negative bacteria participates in diverse virulence phenotypes (module III) and *ag43*, autoaggregation (module IV).

References

1. **Blom M, Meyer A, Gerner-Smidt P, Gaarslev K, Espersen F**. 1999. Evaluation of Statens Serum Institut enteric medium for detection of enteric pathogens. J Clin Microbiol **37**:2312-6.,
2. **Willshaw GA, Smith HR, Scotland SM, Rowe B**. 1985. Cloning of genes determining the production of vero cytotoxin by *Escherichia coli*. J Gen Microbiol **131**:3047-3053.
3. **Thomas A, Smith HR, Willshaw GA, Rowe B**. 1991. Non-radioactively labelled polynucleotide oligonucleotide DNA probes for selectively detecting *Escherichia coli* strains producing vero cytotoxins VT1, VT2 and VT2 variant. Mol Cell Probes **5**:129-135.
4. **Venkatesan MM, Buysse JM, Kopecko DJ**. 1989. Use of *Shigella flexneri ipaC* and *ipaH* gene sequences for the general identification of *Shigella* spp. and enteroinvasive *Escherichia coli*. J Clin Microbiol **27**:2687-2691.
5. **Jerse AE, Yu J, Tall BD, Kaper JB**. 1990. A genetic locus of enteropathogenic *Escherichia coli* necessary for the production of attaching and effaing lesions on tissue culture cells. Proc Natl Acad Sci USA **87**:7839-7843.
6. **Sommerfelt H, Kalland KH, Raj P, Moseley SL, Bhan MK, Bjorvatn B**. 1988. Cloned polynucleotide and synthetic oligonucleotide probes used in colony hybridization are equally efficient in the identification of enterotoxigenic *Escherichia coli* [published erratum appears in J Clin Microbiol 1990 Mar;28(3):642]. J Clin Microbiol **26**:2275-2278.
7. Scheutz F. 1997. Vero cytotoxin producing *Escherichia coli* (VTEC) isolated from Danish patients. Ph. D. thesis. Statens Serum Intitut's own print.
8. **Persson S, Olsen KEP, Scheutz F, Krogfelt KA, Gerner-Smidt P**. 2007. A method for fast and simple detection of major diarrhoeagenic *Escherichia coli* in the routine diagnostic laboratory. Clinical Microbiology and Infection **13**:516-524. https://doi.org/10.1111/j.1469-0691.2007.01692.x
9. **Scheutz F, Teel LD, Beutin L, Pierard D, Buvens G, Karch H, Mellmann A, Caprioli A, Tozzoli R, Morabito S, Strockbine NA, Melton-Celsa AR, Sanchez M, Persson S, O'Brien AD**. 2012. Multicenter evaluation of a sequence-based protocol for subtyping shiga toxins and standardizing Stx nomenclature. J Clin Microbiol **50**:2951-2963. https://doi.org/10.1128/JCM.00860-12
10. **Iguchi A, Iyoda S, Seto K, Morita-Ishihara T, Scheutz F, Ohnishi M**. 2015. *Escherichia coli* O-genotyping PCR; a comprehensive and practical platform for molecular O-

serogrouping. J Clin Microbiol doi:JCM.00321-15 [pii];10.1128/JCM.00321-15 [doi]. JCM.00321-15 [pii];10.1128/JCM.00321-15 [doi]

11. **Kjaeldgaard P, Nissen B, Lange N, Laursen H**. 1986. Evaluation of Minibact, a new system for rapid identification of *Enterobacteriaceae*. Comparison of Minibact, Micro-ID and API 20E with a conventional method as reference. Acta Pathol Microbiol Immunol Scand **94**:57-61.
12. Lautrop H, Høiby N, Bremmelgaard A, Korsager B. 1979. Bakteriologiske undersøgelsesmetoder, 1 ed. FADL's Forlag, København.
13. **Ørskov F, Ørskov I**. 1984. Serotyping of *Escherichia coli*. Meth Microbiol **14**:43-112.
14. **Scheutz F, Cheasty T, Woodward D, Smith HR**. 2004. Designation of O174 and O175 to temporary O groups OX3 and OX7, and six new *E. coli* O groups that include Verocytotoxin-producing *E. coli* (VTEC): O176, O177, O178, O179, O180 and O181. APMIS **112**:569-584.
15. **Beutin L, Montenegro MA, Ørskov I, Ørskov F, Prada J, Zimmerman S, Stephan R**. 1989. Close association of verotoxin (shiga-like toxin) production with enterohemolysin production in strains of *Escherichia coli*. J Clin Microbiol **27**:2559-2564.
16. **Levine MM, Xu J-G, Kaper JB, Lior H, Prado V, Nataro JP, Karch H, Wachsmuth IK**. 1987. A DNA probe to identify enterohemorrhagic *Escherichia coli* of O157:H7 and other serotypes that cause hemorrhagic colitis and hemolytic uremic syndrome. J Infect Dis **156**:175-182.
17. **Small PLC, Falkow S**. 1986. Development of a DNA Probe for the Virulence Plasmid of *Shigella* spp. and Enteroinvasive *Escherichia coli*, p 121-124. *In* Leive L, Bonventre PF, Morello JA, Silver SD, Wu WC, Adam A (ed), Microbiology.
18. **Baudry B, Savarino SJ, Vial P, Kaper JB, Levine MM**. 1990. A sensitive and specific DNA probe to identify enteroaggregative *Escherichia coli*, a recently discovered diarrheal pathogen. J Infect Dis **161**:1249-1251. http://www.ncbi.nlm.nih.gov/pubmed/2189007?dopt=Citation
19. **Bilge SS, Clausen CR, Lau W, Moseley SL**. 1989. Molecular characterization of a fimbrial adhesin F1845, mediating diffuse adherence of diarrhea-associated *Escherichia coli* to HEp-2 cells. J Bacteriol **171**:4281-4289.
20. **Paton AW, Srimanote P, Woodrow MC, Paton JC**. 2001. Characterization of Saa, a novel autoagglutinating adhesin produced by locus of enterocyte effacement-negative shiga-toxigenic *Escherichia coli* strains that are virulent for humans. Infection and Immunity **69**:6999-7009. http://www.ncbi.nlm.nih.gov/pubmed/11598075
21. **Joensen KG, Tetzschner AM, Iguchi A, Aarestrup FM, Scheutz F**. 2015. Rapid and easy *in silico* serotyping of *Escherichia coli* using whole genome sequencing (WGS) data. J Clin Microbiol **53**:2410-2426. 10.1128/JCM.00008-15 [doi]
22. **Malberg Tetzschner AM, Johnson JR, Johnston BD, Lund O, Scheutz F**. 2020. *In Silico* Genotyping of *Escherichia coli* Isolates for Extraintestinal Virulence Genes by Use of Whole-Genome Sequencing Data. J Clin Microbiol **58**. 10.1128/JCM.01269-20
23. **Scheutz F, Hald Nielsen C, von Mentzer A**. 2024. Construction of the ETECFinder database for the characterization of enterotoxigenic *Escherichia coli* (ETEC) and revision of the VirulenceFinder web tool at the CGE website. Journal of Clinical Microbiology doi:10.1128/jcm.00570-23. 10.1128/jcm.00570-23
24. **Joensen KG, Scheutz F, Lund O, Hasman H, Kaas RS, Nielsen EM, Aarestrup FM**. 2014. Real-time whole-genome sequencing for routine typing, surveillance, and outbreak detection of verotoxigenic *Escherichia coli*. J Clin Microbiol **52**:1501-10. 10.1128/JCM.03617-13

25. **Montero DA, Velasco J, Del CF, Puente JL, Padola NL, Rasko DA, Farfan M, Salazar JC, Vidal R**. 2017. Locus of Adhesion and Autoaggregation (LAA), a pathogenicity island present in emerging Shiga Toxin-producing *Escherichia coli* strains. Sci Rep **7**:7011. 10.1038/s41598-017-06999-y [doi];10.1038/s41598-017-06999-y [pii]
